# Trends and hospital practice variation for analgesia for children with sickle cell disease with vaso-occlusive pain episodes: An 11-year analysis

**DOI:** 10.1016/j.ajem.2024.10.028

**Published:** 2024-10-13

**Authors:** Mohamed Eltorki, Matt Hall, Sriram Ramgopal, Pradip P. Chaudhari, Oluwakemi Badaki-Makun, Chris A. Rees, Kelly R. Bergmann, Daniel J. Shapiro, Frank Gonzalez, Timothy Phamduy, Mark I. Neuman

**Affiliations:** a Section of Pediatric Emergency Medicine, Department of Pediatrics, Cumming School of Medicine, Alberta Children’s Hospital Research Institute, University of Calgary, 28 Oki Drive NW, Calgary, Alberta T3B 6A8, USA; b Division of Pediatric Emergency Medicine, Department of Pediatrics, Faculty of Health Sciences, McMaster University, 1200 Main St W, Hamilton, Ontario L8S 4L8, Canada; c Children’s Hospital Association, 6803 W 64th St, Overland Park, Kansas 66202, USA; d Division of Emergency Medicine, Department of Pediatrics, Ann & Robert H. Lurie Children’s Hospital of Chicago, Northwestern University Feinberg School of Medicine, Ann & Robert H. Lurie Children’s Hospital, 225 E Chicago Ave, Chicago, IL 60611, USA; e Division of Emergency and Transport Medicine, Children’s Hospital Los Angeles, Department of Pediatrics, Keck School of Medicine of the University of Southern California, Children’s Hospital Los Angeles, 4650 Sunset Blvd, Los Angeles, California 90027, USA; f Division of Pediatric Emergency Medicine, Department of Pediatrics, Johns Hopkins University School of Medicine, Center for Data Science in Emergency Medicine, Johns Hopkins University, Johns Hopkins Hospital, 1800 Orleans St, Baltimore, MD 21287, USA; g Division of Pediatric Emergency Medicine, Emory University School of Medicine, Children’s Healthcare of Atlanta at Egleston, 1405 Clifton Rd NE, Atlanta, GA 30322, USA; h Department of Emergency Medicine, Children’s Minnesota, 915 E 25th St, Minneapolis, MN, USA; i Division of Pediatric Emergency Medicine, University of California, UCSF Benioff Children’s Hospital, 1825 Fourth St, San Francisco, California 94158, USA; j Division of Pediatric Emergency Medicine, Dayton Children’s Hospital, Department of Pediatrics, Wright State University, Dayton Children’s Hospital, 1 Children’s Plaza, Dayton, OH 45404, USA; k Division of Emergency Medicine, Boston Children’s Hospital, 300 Longwood Ave, Boston, MA 02115, USA

**Keywords:** Pediatric emergency medicine, Pain management, Hospital practice patterns, Vaso-occlusive crisis, Analgesic trends, Multimodal analgesia, Opioid use, Non-steroidal anti-inflammatory drugs (NSAIDs)

## Abstract

This cross-sectional analysis of 86,111 visits for sickle cell disease and vaso-occlusive episodes (VOE) in U.S. pediatric emergency departments between 2013 and 2023 shows increased use of NSAIDs, ketamine, and acetaminophen, with unchanged opioid use. Hospitals with a higher volume of VOE visits more frequently administered opioids.

**Background:**

Vaso-occlusive episodes (VOEs) are a hallmark of sickle cell disease (SCD), leading to frequent emergency department (ED) visits. Effective pain management is crucial, with guidelines recommending routine use of non-steroidal anti-inflammatory drugs (NSAIDs) with opioids, and emerging evidence supporting ketamine use. However, these recommendations are based on low-certainty evidence, and the impact of these guidelines on analgesia use over time remains unclear.

**Objective:**

This study aimed to analyze trends in analgesia use over an 11-year period in pediatric SCD patients presenting to U.S. EDs with VOE and assess variations in treatment across hospitals.

**Methods:**

A cross-sectional study was conducted using data from the Pediatric Health Information System covering 34 U.S. children’s hospitals from January 1, 2013, to December 31, 2023. The primary outcomes were the proportions of visits where opioids, NSAIDs, acetaminophen, and/or ketamine were administered on the first calendar day of the initial visit. Secondary outcomes included the co-administration of NSAIDs with opioids. Logistic and linear regression models were used to assess trends and hospital-level variations.

**Results:**

A total of 86,111 ED visits for VOE were analyzed. Opioids were administered in 82 % of encounters, NSAIDs in 72 %, acetaminophen in 17 %, and ketamine in 1 %. Co-administration of NSAIDs with opioids occurred in 59 % of the visits. Among discharged patients, there was a positive trend for NSAID use (slope: 1.68 %/year, 95 % CI: 0.91 %, 2.45 %) and NSAID-opioid co-administration (slope: 1.03 %/year, 95 % CI: 0.37 %, 1.69 %) over time. Acetaminophen use also increased over the study period (slope: 0.99 %/year, 95 % CI: 0.80 %, 1.17 %). In hospitalized patients, there was a significant upward trend for acetaminophen (slope: 1.29 %/year, 95 % CI: 0.69 %, 1.89 %) and ketamine (slope: 0.36 %/year, 95 % CI: 0.27 %, 0.45 %), while opioid use remained unchanged. Significant hospital-level variations were observed, with larger hospitals more likely to administer opioids but less likely to co-administer NSAIDs with opioids compared to medium-volume hospitals.

**Conclusion:**

Over the past decade, the use of NSAIDs, acetaminophen, and ketamine has increased in the management of VOE in pediatric SCD patients, while opioid use remains consistent. The co-administration of NSAIDs and opioids has also increased, reflecting guideline adherence. Variations in analgesia practices across hospitals underscore the need for standardizing pain management strategies in this population.

## 1. Background

Vaso-occlusive episodes (VOEs) are a defining feature of sickle cell disease (SCD), accounting for 75 % of all SCD-related emergency department (ED) visits [1]. Effective pain management is critical for patients experiencing VOE in SCD, as these episodes are associated with significant morbidity [2]. The American Society of Hematology (ASH) [3] a nd the National Heart, Lung, and Blood Institute (NHLBI) [4] recommend use of non-steroidal anti-inflammatory drugs (NSAIDs) for all patients with VOE pain, and rapid administration of opioids, taking into account factors such as prior response to specific potencies and doses of opioids. Furthermore, the use of ketamine has been recommended for hospitalized children, supported by few trials showing its non-inferiority to morphine [3,5,6]. However, all these analgesia recommendations in the guidelines were based on low-certainty evidence, with several systematic reviews demonstrating mixed and inconclusive results [7,8], raising questions about whether analgesia use over time has been influenced by these guidelines.

Our objectives were to: 1) analyze trends over an 11-year period ending in 2023 for commonly used analgesia in children with SCD presenting to a U.S. ED with VOE, and 2) assess variations in hospital treatment of VOE. By understanding these trends and variations, we aim to gain valuable insights into the effectiveness of current guidelines and identify gaps in care. This knowledge has the potential to inform future research and guideline development, ultimately leading to improved pain management strategies and better outcomes for these patients.

## 2. Methods

We performed a cross-sectional study of ED encounters for patients with a VOE from 34 US children’s hospitals who provide data to the Pediatric Health Information System (PHIS). PHIS utilizes de-identified administrative data on ED and inpatient visits and is managed by the Children’s Hospital Association (Overland Park, Kansas) [9]. The study followed STROBE guidelines [10] and was exempt from local ethics review.

### 2.1. Cohort definition

We included all visits of patients aged 0 to 19 years to a PHIS hospital between January 1, 2013, and December 31, 2023, with a primary or non-primary diagnosis of a VOE using the International Classification of Diseases, 9th and 10th revisions diagnosis codes (supplement). We excluded encounters of patients transferred from another hospital or those with in-hospital mortality.

### 2.2. Outcomes

The primary outcomes were the proportions of visits in which opioids, NSAIDs, acetaminophen and/or ketamine were administered on the first calendar day of the initial visit. The secondary outcome was the proportion of children who were co-administered NSAIDs and opioids (i.e., patient visits in which NSAIDs were given, when opioids were used for pain relief). This outcome was chosen since both 2014 NHLBI guidelines and the 2018 ASH guidelines recommend NSAIDs in addition to opioids for VOEs [3]. The PHIS database provides a comprehensive list of Multum Lexicon drug codes corresponding to medications administered during the visit (supplement). We included all relevant codes for opioids, NSAIDs, acetaminophen, and ketamine across all routes of administration. Additionally, we abstracted demographic data (age, sex, race, primary payer), other diagnoses related to SCD (i.e., acute chest syndrome, splenic sequestration, cerebral vascular involvement) and non-hematologic chronic complex conditions [11].

### 2.3. Analysis

We stratified our analysis by disposition and by hospital. We used a linear regression model for each medication type to assess time trends, with the year of the visit as the independent variable and the proportion of visits receiving the medication as the dependent variable. Hospitals were categorized into small, medium, and large volume based on tertiles of the number of VOEs treated during each study period, rather than the overall hospital size. This approach reflects the specific experience of each hospital in managing sickle cell disease, where the volume of VOEs treated is a more relevant indicator of experience than the hospital’s total number of patients seen. Using percentiles, hospitals were divided into three groups: 11 hospitals were classified as small-volume (209 to 999 VOEs), 11 as medium-volume (1176 to 2507 VOEs), and 12 as large-volume (2637 to 15,251 VOEs). We calculated odds ratios (OR) with 95 % confidence intervals (CI) using logistic regression to compare the likelihood of receiving opioids and combination opioids and NSAIDs among hospital size groups. All statistical tests were 2 sided, and *P* values <0.05 were considered statistically significant. Analyses were performed by using SAS software version 9.4 (Statistical Analysis System Institute, Inc., Cary, NC), and trends were visualized using line plots generated with the matplotlib library in Python (version 3.8).

## 3. Results

There were 86,111 ED visits for VOE during the study period, and adolescents 15 years and older accounted for 37,294 (43 %) of the visits. Overall, 44,037 (51.1 %) were males, and 78,897 (92 %) were non-Hispanic Black. Visit numbers were stable over time (*p* = 0.540) and 48,149 (56 %) of visits resulted in hospitalization (Table 1).

### 3.1. Trends in the use of analgesics

**Fig. 1.**
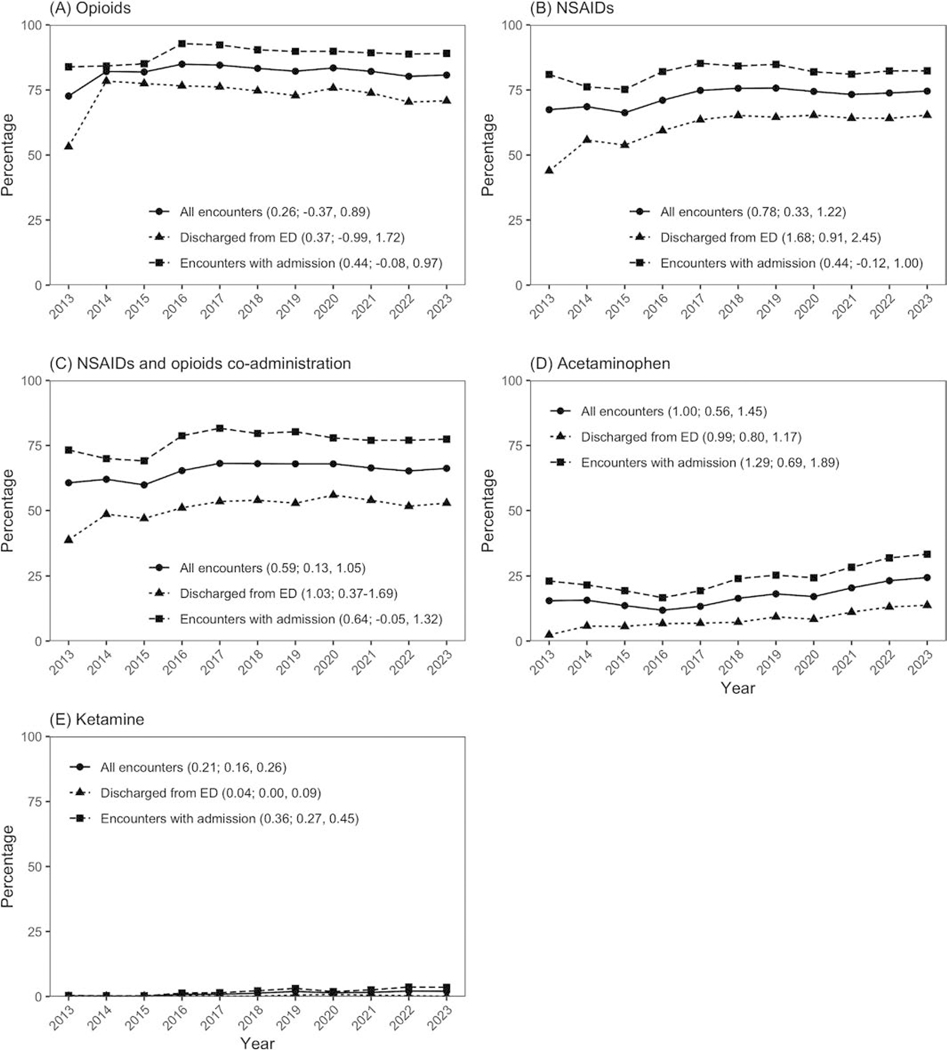
Trend lines for opioids, Non-Steroidal-Anti Inflammatory-Drugs (NSAIDs), opioids-NSAID combination therapy, acetaminophen and ketamine.

The most frequently used analgesics were opioids, which were administered in 70,283 (82 %) encounters, followed by NSAIDs in 62,204 (72 %), acetaminophen in 14,871 (17 %), and ketamine in 990 (1 %). Among discharged patients, there was a positive trend for NSAIDs use (slope: 1.68 %/year, 95 % CI: 0.91 % to 2.45 %), NSAID co-administration with opioids (slope: 1.03 %/year, 95 % CI: 0.37 % to 1.69 %), and acetaminophen use (slope: 0.99 %/year, 95 % CI: 0.80 % to 1.17 %) over time. Among admitted patients, there was a significant upward trend for acetaminophen (slope: 1.29 %/year 95 % CI: 0.69 % to 1.89 %) and ketamine (slope: 0.36 %/year 95 % CI: 0.27 %, 0.45 %), but there were no significant changes for NSAIDs or NSAIDs co-administration with opioids. Among admitted and discharged patients, there was no change in the use of opioids over time (Fig. 1).

### 3.2. Hospital-level variation

The frequency of opioid administration varied across hospitals, ranging from 52.3 % to 90.3 % with a median of 81.8 % (interquartile range [IQR] 79.0 %, 86.2 %). Similarly, the frequency of NSAID use among patients who received opioids also showed considerable variation, ranging from 14.7 % to 93.5 % with a median of 82 % (IQR 77.8 %, 89.2 %).

**Fig. 2.**
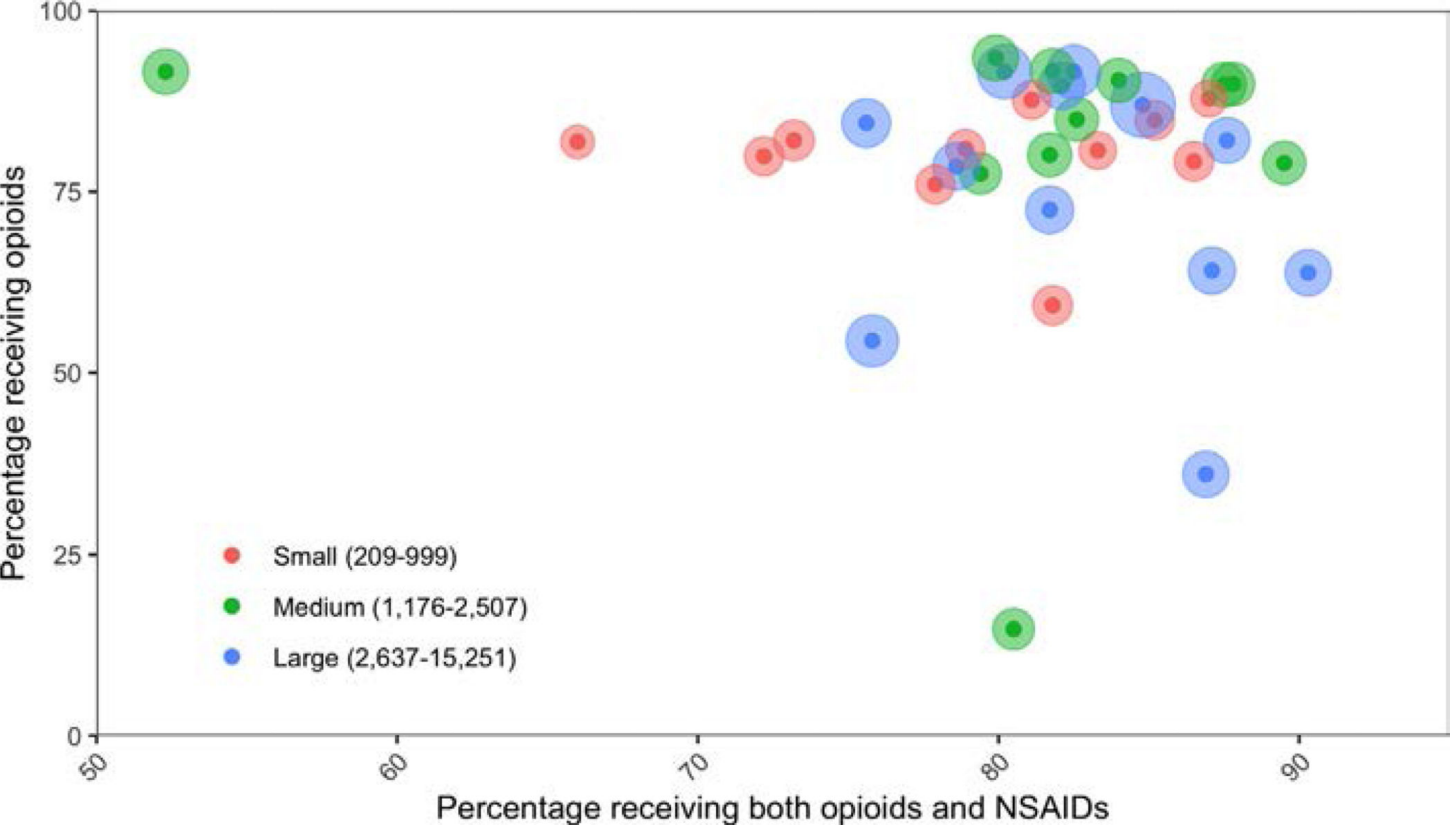
Scatter plot for Hospital Variation in opioid and non-steroidal anti-inflammatory drugs (NSAIDs) and opioid co-administration.

Large-volume hospitals were more likely to administer opioids compared to small-volume hospitals (OR: 1.20, 95 % CI: 1.13 to 1.28) and medium-volume hospitals (OR: 1.20, 95 % CI: 1.15 to 1.24). There was no difference in the frequency of opioid administration between small-volume and medium-volume hospitals (OR: 1.01, 95 % CI: 0.94 to 1.08). Large-volume hospitals were less likely to co-administer NSAIDs with opioids than medium-volume hospitals (OR: 0.74, 95 % CI: 0.71 to 0.78) but not compared to small volume hospitals (OR: 0.97, 95 % CI: 0.91 to 1.04). Medium hospitals were more likely to co-administer NSAIDs with opioids than small-volume hospitals (OR: 1.31, 95 % CI: 1.21 to 1.41) (Fig. 2).

## 4. Discussion

In this 11-year cross-sectional study of 34 children’s hospitals, the use of NSAIDs alone or in combination with opioids, acetaminophen, and ketamine all had an upward trend in use, whereas opioid use was unchanged. The use of ketamine, though rare, increased 9fold during the study period, primarily among hospitalized children, indicating a growing recognition of its use in managing severe pain. The variability in opioid administration across hospitals, with large-volume hospitals being more likely to use opioids and less likely to use NSAIDs in combination, highlights differences in clinical practice and resource availability and underscores opportunities to standardize care.

Our analysis revealed that patients discharged from the ED were more likely to receive NSAIDs alone or in combination with opioids, which is consistent with recent ASH and NHLBI guideline updates [3,4]. These emphasize the importance of multimodal analgesia, aiming to minimize opioid use while ensuring effective pain management. The stable use of opioids, despite the upward trend in NSAID use, indicates that opioids remain a cornerstone of VOE pain management. This may reflect their established role in providing immediate relief for acute pain episodes in SCD.

The higher utilization of opioids in larger-volume hospitals could be attributed to greater familiarity and confidence in managing severe VOE pain in these settings. Larger-volume hospitals may also have more resources and protocols in place to standardize pain treatment, while providing adequate monitoring and management of potential opioid-related complications. Smaller-volume hospitals may be impeded by lack of those resources and some of the known barriers to using opioids in patients with SCD, such as fear of drug abuse, and skepticism in patients’ report of pain severity among clinicians and nurses, [12] and beliefs that opioid addiction frequently develops in patients with SCD [13]. This has not been supported by empirical evidence, with a recent study showing that SCD is not a risk factor for opioid use disorders relative to other chronic conditions [14].

Our multicenter study builds upon previous local evidence evaluating long-term trends of pharmacologic treatment for patients with SCD. Prior single-center retrospective chart reviews have suggested that opioid use ranges from 70 to 80 % for treatment of VOE [15,16]. After publication of the NHLBI guidelines, a cross sectional analysis of data from 7 Pediatric Emergency Care Applied Research Network sites over a 3-year period including 4578 visits was published [17]. This analysis focused on the time to opioid administration but did not provide data on the frequencies of different analgesics used [17]. They found poor adherence to timely management of pain with opioids with only half of the visits classified as guideline congruent (receiving opioid within 60-min of arrival) [17]. These findings, highlight the need for ongoing efforts to standardize pain management practices across pediatric EDs, ensuring that all children with SCD receive equitable care regardless of the hospital they visit.

### 4.1. Limitations

The retrospective design and reliance on an administrative database restrict our ability to adjust for treatment predictors such as pain severity, pre-hospital (EMS or home) treatments administered, socioeconomic status, or specific clinical protocols in different hospitals. Additionally, the de-identified nature of the PHIS database limits our capacity to conduct detailed patient-level analyses, which could provide deeper insights into individual treatment responses and outcomes. Our definition of first day of treatment (calendar day) may have resulted into an under-estimation of the proportions of patients receiving analgesia if patients presented late in the evening. All hospitals included in this study are tertiary care pediatric hospitals, and thus the findings may not reflect practice in general EDs. Furthermore, the exclusion of patients transferred from other hospitals or those with in-hospital mortality might introduce selection bias, potentially underestimating the severity of cases managed in smaller hospitals.

## 5. Conclusion

Our study provides valuable insights into the evolving practice patterns of analgesia use for VOE in children with SCD across U.S. children’s hospitals. While the use of NSAIDs, acetaminophen, and ketamine has increased, opioids use has not changed and remain a critical component of VOE pain management. The observed variations in analgesia practices highlight the need for ongoing efforts to standardize and optimize pain management strategies in this vulnerable population.

## Supplementary Material

Supplementary Materials

## Figures and Tables

**Table 1:** Study cohort (*N* = 86,111): any diagnosis of vaso-occlusive episode.

Characteristic	ED visits N (%)	Discharged N (%)	Hospitalized N (%)

N	86,111	37,962 (44.1)	48,149 (55.9)
Age (years) <1	1445 (1.7)	631 (1.7)	814 (1.7)
1–4	9639 (11.2)	5687 (15.0)	3952 (8.2)
5–9	16,216 (18.8)	9120 (24.0)	7096 (14.7)
10–14	21,517 (25.0)	8788 (23.1)	12,729 (26.4)
15–18	26,056 (30.3)	9344 (24.6)	16,712 (34.7)
19–20	11,238 (13.1)	4392 (11.6)	6846 (14.2)
Sex: Male	44,037 (51.1)	19,947 (52.5)	24,090 (50.0)
Race and Ethnicity Non-Hispanic White	1093 (1.3)	481 (1.3)	612 (1.3)
Non-Hispanic Black	78,897 (91.6)	34,680 (91.4)	44,217 (91.8)
Hispanic	4079 (4.7)	1647 (4.3)	2432 (5.1)
Non-Hispanic Asian	171 (0.2)	83 (0.2)	88 (0.2)
Other	1871 (2.2)	1071 (2.8)	800 (1.7)
Payor Government	63,263 (73.5)	27,452 (72.3)	35,811 (74.4)
Private	18,955 (22.0)	8412 (22.2)	10,543 (21.9)
Other	3893 (4.5)	2098 (5.5)	1795 (3.7)
Any Non-Hematologic Complex Chronic Condition	8713 (10.1)	915 (2.4)	7798 (16.2)
Weekend ED encounter	22,190 (25.8)	9886 (26.0)	12,304 (25.6)
Acute chest syndrome	4333 (5.0)	153 (0.4)	4180 (8.7)
Splenic sequestration	809 (0.9)	28 (0.1)	781 (1.6)
Cerebral vascular involvement	8 (0.0)	0 (0.0)	8 (0.0)
Year 2013	7753 (9.0)	2829 (7.5)	4924 (10.2)
2014	8410 (9.8)	3125 (8.2)	5285 (11.0)
2015	8480 (9.8)	3544 (9.3)	4936 (10.3)
2016	7691 (8.9)	3729 (9.8)	3962 (8.2)
2017	7942 (9.2)	3813 (10)	4129 (8.6)
2018	7700 (8.9)	3477 (9.2)	4223 (8.8)
2019	8034 (9.3)	3607 (9.5)	4427 (9.2)
2020	6273 (7.3)	2844 (7.5)	3429 (7.1)
2021	7377 (8.6)	3403 (9.0)	3974 (8.3)
2022	8067 (9.4)	3758 (9.9)	4309 (8.9)
2023	8384 (9.7)	3833 (10.1)	4551 (9.5)
Drugs Administered Opioids	70,283 (81.6)	27,706 (73.0)	42,577 (88.4)
NSAIDs	62,204 (72.2)	23,070 (60.8)	39,134 (81.3)
Opioids and NSAIDs combination	56,085 (65.1)	19,391 (51.1)	36,694 (76.2)
Acetaminophen	14,871 (17.3)	3185 (8.4)	11,686 (24.3)
Ketamine	990 (1.1)	109 (0.3)	881 (1.8)

## Appendix A. Supplementary data

Supplementary data to this article can be found online at https://doi.org/10.1016/j.ajem.2024.10.028.

## Abbreviations:

VOE: Vaso-occlusive episodes
SCD: Sickle cell disease
NSAIDs: Non-steroidal anti-inflammatory drugs
ED: Emergency department
ASH: American Society of Hematology
NHLBI: National Heart, Lung, and Blood Institute
PHIS: Pediatric Health Information System
STROBE: Strengthening the Reporting of Observational Studies in Epidemiology
OR: Odds ratio
CI: Confidence interval
IQR: Interquartile range
SAS: Statistical Analysis System (software)

## References

[1] Lanzkron S, Carroll CP, Haywood C Jr. The burden of emergency department use for sickle-cell disease: an analysis of the national emergency department sample database. Am J Hematol. 2010;85(10):797–9. 10.1002/ajh.21807. 20730795 PMC3431910

[2] Bailey M, Abioye A, Morgan G, Burke T, Disher T, Brown S, Relationship between Vaso-occlusive crises and important complications in sickle cell disease patients. Blood. 2019;134(Supplement_1):2167. 10.1182/blood-2019131721.

[3] Brandow AM, Carroll CP, Creary S, Edwards-Elliott R, Glassberg J, Hurley RW, American Society of Hematology 2020 guidelines for sickle cell disease: management of acute and chronic pain. Blood Adv. 2020;4(12):2656–701. 10.1182/bloodadvances.2020001851. PMC7322963 32559294

[4] Yawn BP, Buchanan GR, Afenyi-Annan AN, Ballas SK, Hassell KL, James AH, Management of sickle cell disease: summary of the 2014 evidence-based report by expert panel members. JAMA. 2014;312(10):1033–48. 10.1001/jama.2014.10517. 25203083

[5] Lubega FA, DeSilva MS, Munube D, Nkwine R, Tumukunde J, Agaba PK, Low dose ketamine versus morphine for acute severe vaso occlusive pain in children: a randomized controlled trial. Scand J Pain. 2018;18(1):19–27. 10.1515/sjpain-2017-0140. 29794277

[6] Alshahrani MS, AlSulaibikh AH, ElTahan MR, AlFaraj SZ, Asonto LP, AlMulhim AA, Ketamine administration for acute painful sickle cell crisis: a randomized controlled trial. Acad Emerg Med. 2022;29(2):150–8. 10.1111/acem.14382. 34449939 PMC9292870

[7] Lowe M, Bambhroliya Z, Patel H, Patel VJ, Vudugula SA, Cheruvu NP, Emerging therapies for the Management of Pain and Vaso-Occlusive Crises in patients with sickle cell disease: a systematic review of randomized controlled trials. Cureus. 2023;15(4):e38014. 10.7759/cureus.38014. PMC10204617 37223201

[8] Cooper TE, Hambleton IR, Ballas SK, Johnston BA, Wiffen PJ. Pharmacological interventions for painful sickle cell vaso-occlusive crises in adults. Cochrane Database Syst Rev. 2019;2019(11). 10.1002/14651858.CD012187.pub2. PMC6863096 31742673

[9] Pediatric Health Information System. Children’s hospital association. Available at. https://www.childrenshospitals.org/; 2024 August 20.

[10] von Elm E, Altman DG, Egger M, Pocock SJ, Gotzsche PC, Vandenbroucke JP, The strengthening the reporting of observational studies in epidemiology (STROBE) statement: guidelines for reporting observational studies. J Clin Epidemiol. 2008; 61(4):344–9. 10.1016/j.jclinepi.2007.11.008. 18313558

[11] Feudtner C, Feinstein JA, Zhong W, Hall M, Dai D. Pediatric complex chronic conditions classification system version 2: updated for ICD-10 and complex medical technology dependence and transplantation. BMC Pediatr. 2014;14:199. 10.1186/1471-2431-14-199. 25102958 PMC4134331

[12] Pack-Mabien A, Labbe E, Herbert D, Haynes J Jr. Nurses’ attitudes and practices in sickle cell pain management. Appl Nurs Res. 2001;14(4):187–92. 10.1053/apnr.2001.26783. 11699021

[13] Zempsky WT. Evaluation and treatment of sickle cell pain in the emergency department: paths to a better future. Clin Pediatr Emerg Med. 2010;11(4):265–73. 10.1016/j.cpem.2010.09.002. 21499553 PMC3076949

[14] Jonassaint CR, O’Brien J, Nardo E, Feldman R, Stanton M, DeCastro L, Prevalence of substance use disorders in sickle cell disease compared to other chronic conditions: a population-based study of black American adults. J Gen Intern Med. 2023; 38(5):1214–23. 10.1007/s11606-022-07786-w. 36220945 PMC10110804

[15] Zempsky WT, Loiselle KA, McKay K, Lee BH, Hagstrom JN, Schechter NL. Do children with sickle cell disease receive disparate care for pain in the emergency department? J Emerg Med. 2010;39(5):691–5. 10.1016/j.jemermed.2009.06.003. 19703740

[16] Frei-Jones MJ, Baxter AL, Rogers ZR, Buchanan GR. Vaso-occlusive episodes in older children with sickle cell disease: emergency department management and pain assessment. J Pediatr. 2008;152(2):281–5. 10.1016/j.jpeds.2007.06.040. 18206703 PMC2359225

[17] Brousseau DC, Alpern ER, Chamberlain JM, Ellison AM, Bajaj L, Cohen DM, A multiyear cross-sectional study of guideline adherence for the timeliness of opioid administration in children with sickle cell pain crisis. Ann Emerg Med. 2020;76 (3S):S6–S11. 10.1016/j.annemergmed.2020.08.006. 32928464 PMC8689682

