# Supplementary material for: Trends and hospital practice variation for analgesia for children with sickle cell disease with vaso-occlusive pain episodes: An 11-year analysis
Source: Am J Emerg Med. Author manuscript; Available in PMC 2026 Feb 22. (PMC12925324; doi:10.1016/j.ajem.2024.10.028)
Supplement: Supplementary Materials [file NIHMS2044189-supplement-Supplementary_Materials.docx]

# Supplemental Information

## Sickle Cell Diagnosis Codes (GIST Sheet)

| Code | Description | Group |
| --- | --- | --- |
| D57418 | Sickle-cell Thalassemia, Unspecified, With Crisis With Other Specified Complication | Crisis |
| D57419 | Sickle-cell Thalassemia, Unspecified, With Crisis | Crisis |
| D5709 | Hb-ss Disease With Crisis With Other Specified Complication | Crisis |
| D5700 | Hb-ss Disease With Crisis, Unspecified | Crisis |
| D57218 | Sickle-cell/hb-c Disease With Crisis With Other Specified Complication | Crisis |
| D57219 | Sickle-cell/hb-c Disease With Crisis, Unspecified | Crisis |
| D57818 | Other Sickle-cell Disorders With Crisis With Other Specified Complication | Crisis |
| D57819 | Other Sickle-cell Disorders With Crisis, Unspecified | Crisis |
| D57459 | Hb-S or SS beta plus with crisis | Crisis |
| D57439 | Hb-S or SS beta zero with crisis | Crisis |
| 28242 | Sickle-cell thalassemia with crisis | Crisis |
| 28262 | Hb-ss disease with crisis | Crisis |
| 28264 | Sickle-cell/hb-c disease with crisis | Crisis |
| 28269 | Other sickle-cell disease with crisis | Crisis |

## Pain Medication Group Codes (Fig 1 Sheet)

### Opiates

| Medication | Code |
| --- | --- |
| Fentanyl (base) (citrate) | 112115 |
| Hydromorphone HCL | 112117 |
| Morphine Sulfate | 112131 |
| Oxycodone HCL | 112135 |
| Oxymorphone HCK | 112137 |
| Narcotic analgesic combinations* | 112158 |
| Codeine (phosphate) (sulfate) | 112113 |
| Tramadol HCL | 112228 |
| Meperidine HCL | 112123 |
| Buprenorphine HCL | 112162 |
| Buprenorphine HCl/Naloxone HCl | 119177 |
| Methadone | 112127 |
| Naltrexone HCL | 119181 |
| Naloxone HCL | 119175 |
| New Codes |  |
| Narcotic analgesics unspecified | 112100 |
| Alfentanil HCl | 112111 |
| Hydrocodone bitartrate | 112114 |
| Levorphanol tartrate | 112123 |
| Morphine sulfate and naltrexone HCl | 112132 |
| Opium | 112133 |
| Propoxyphene (dextropropoxyphene) (HCl) (napsylate) | 112141 |
| Remifentanil HCl | 112143 |
| Sufentanil citrate | 112145 |
| Tapentadol | 112146 |
| Narcotic analgesic and aspirin combinations | 112148 |
| Narcotic analgesic combinations | 112158 |
| Butorphanol tartrate | 112161 |
| Nalbuphine HCl | 112163 |
| Pentazocine (HCl) (lactate) | 112164 |
| Dezocine | 112165 |
| Pentazocine combinations | 112168 |

### NSAIDs

| Medication | Code |
| --- | --- |
| Ketorolac tromethamine | 112270 |
| Ibuprofen | 112260 |
| Naproxen (acid) (sodium) | 112288 |
| Diclofenac | 112310 |
| New Codes |  |
| Nonnarcotic analgesics unspecified | 112200 |
| Aspirin (acetylsalicylic acid) (ASA) | 112205 |
| Aspirin (acetylsalicylic acid) (ASA) buffered | 112209 |
| Choline salicylate | 112211 |
| Diflunisal | 112214 |
| Magnesium salicylate | 112217 |
| Salsalate (salicylsalicylic acid) | 112220 |
| Sodium salicylate | 112224 |
| Sodium thiosalicylate | 112226 |
| Diclofenac sodium and misoprostol | 112234 |
| Aspirin and other salicylate combinations | 112238 |
| Acetaminophen combinations | 112240 |
| Nonnarcotic analgesic and barbiturate combinations | 112244 |
| Etodolac | 112250 |
| Fenoprofen calcium | 112253 |
| Flurbiprofen (sodium) | 112257 |
| Ketoprofen | 112268 |
| Meclofenamate sodium | 112274 |
| Mefenamic acid | 112278 |
| Meloxicam | 112280 |
| Nabumetone | 112284 |
| Oxaprozin | 112290 |
| Piroxicam | 112294 |
| Sulindac | 112298 |
| Suprofen | 112300 |
| Tolmetin sodium | 112304 |
| Naproxen/lansoprazole | 112312 |
| Naproxen and esomeprazole | 112313 |
| Ziconotide | 112315 |
| Celecoxib | 112351 |
| Rofecoxib | 112352 |
| Valdecoxib | 112353 |
| Other specified non narcotic analgesics | 112499 |

### APAP

| Medication | Code |
| --- | --- |
| Acetaminophen | 112201 |

### Ketamine(s)

| Medication | Code |
| --- | --- |
| Ketamine HCl | 115230 |
| Esketamine | 113273 |

### Magnesium

| Medication | Code |
| --- | --- |
| Magnesium sulphate | 146147 |

### Steroids

| Medication | Code |
| --- | --- |
| Dexamethasone | 154035 |
| Hydrocortisone | 154063 |
| Methylprednisolone | 154067 |
| Prednisone | 154083 |
| Prednisolone | 154081 |
| New codes |  |
| Corticosteroid Unspecified | 154000 |
| Cortisone (acetate) | 154027 |
